# Mechanical endovascular therapy for acute ischemic stroke: An indirect treatment comparison between Solitaire and Penumbra thrombectomy devices

**DOI:** 10.1371/journal.pone.0191657

**Published:** 2018-03-07

**Authors:** Jonathan T. Caranfa, Elaine Nguyen, Rafay Ali, Iregi Francis, Albert Zichichi, Elliott Bosco, Craig I. Coleman, William L. Baker, Christine G. Kohn

**Affiliations:** 1 University of Connecticut School of Medicine, Farmington, Connecticut, United States of America; 2 Idaho State University College of Pharmacy, Meridian, Idaho, United States of America; 3 University of Saint Joseph School of Pharmacy, Hartford, Connecticut, United States of America; 4 University of Connecticut School of Pharmacy, Storrs, Connecticut, United States of America; 5 University of Connecticut School of Pharmacy & UConn/Hartford Hospital Evidence-based Medicine, Hartford, Connecticut, United States of America; 6 University of Connecticut School of Medicine/Hartford Hospital Evidence-based Medicine, Hartford, Connecticut, United States of America; Massachusetts General Hospital, UNITED STATES

## Abstract

**Background:**

Randomized controlled trials (RCTs) have compared mechanical endovascular therapy (MET) in addition to intravenous tissue plasminogen activator (IVtPA) to IVtPA alone for the management of acute ischemic stroke (AIS). Direct comparative studies between individual METs are not available. In lieu of head-to-head randomized control trials, we performed an adjusted indirect treatment comparison (ITC) meta-analysis to assess the comparative efficacy and safety of different METs, Solitaire+IVtPA and Penumbra+IVtPA in AIS patients.

**Methods and findings:**

We searched MEDLINE, the Cochrane Central Register of Controlled Trials and Embase from January 1, 2005 through April 1, 2017 for RCTs in AIS patients, comparing a single MET+IVtPA to IVtPA alone and reporting shift in ordinal modified Rankin Scale (mRS) score at 90 days. Secondary endpoints included 90 day mortality and symptomatic intracranial hemorrhage (sICH). Endpoints were pooled using traditional random effects meta-analysis methods, producing odds ratios and 95% confidence intervals. Adjusted ITCs using pooled estimates were then performed. Three studies (SWIFT PRIME, EXTEND-IA, THERAPY) were included; two evaluating the Solitaire stent retriever and one the Penumbra system. Traditional meta-analysis demonstrated that each MET+IVtPA resulted in increased odds of improving ordinal mRS score vs. IVtPA alone, but did not alter the odds of death or sICH. Adjusted ITC showed no significant difference between the METs for any outcome.

**Conclusion:**

No significant difference in efficacy or safety between the Solitaire and Penumbra devices was observed.

## Introduction

Recent randomized controlled trials (RCTs) have transformed acute ischemic stroke (AIS) treatment by demonstrating that mechanical endovascular therapy (MET) in addition to intravenous tissue plasminogen activator (IVtPA) has superior benefit over IVtPA alone [1–6]. Guidelines recommend MET with a stent retriever as the new standard of care for AIS patients with an occlusion in the intracranial internal carotid artery (ICA) or middle cerebral artery (MCA) [7]. While individual mechanical thrombectomy devices used with IVtPA have demonstrated efficacy in RCTs, no direct head-to-head comparisons exist. To differentiate these devices, we conducted a systematic review and indirect treatment comparison (ITC) to characterize the efficacy and safety of these new mechanical thrombectomy devices for treatment of AIS patients. The two devices analyzed in our study, Solitaire and Penumbra, work by navigating to the intracranial site of occlusion and removing the thrombus. Solitaire employs a stent that expands within the thrombus. The clot is removed by retraction of the stent into a more proximally located micro catheter. Penumbra utilizes a large bore aspiration catheter, which asserts negative pressure suctioning to engage and withdraw the thrombus. In either case, there is restoration of blood flow to the ischemic area.

## Methods

We conducted a systematic literature search in MEDLINE, Cochrane Central Register of Controlled Trials, and Embase from January 1, 2005-April 1, 2017. A previously published search strategy was employed [8]. Manual backward citation tracking was performed to identify additional relevant studies.

We included RCTs that evaluated a single MET device (multiple versions of the same branded device, tested within the same study, were included, i.e. Solitaire Flow Restoration (FR) and Solitaire 2) with IVtPA and assessed a comparator arm of IVtPA in patients with neuroimaging-confirmed AIS who had a proximal anterior circulation intracranial occlusion (in the ICA or MCA: M1 or M2 segment); patients had to present ≤12 hours of symptom onset with a minimum 90-day follow-up. Two investigators determined study eligibility independently, with disagreements resolved by discussion or by a third investigator.

The primary efficacy outcome was shift in ordinal modified Rankin Score (mRS). Secondary outcomes included 90-day mortality and symptomatic intracranial hemorrhage (sICH). Two investigators independently abstracted all data using a common data abstraction tool. The quality of each included RCT was assessed using the Cochrane Risk of Bias tool [E1-2 in S1 Appendix].

Traditional pair-wise meta-analysis was first conducted with events categorized as continuous variables. Analyses were conducted for each pair-wise comparison separately. Weighted averages were reported as odds ratios (ORs) with associated 95% confidence intervals (CIs) using a random-effects model with the Knapp-Hartung estimator [E3 in S1 Appendix]. Sensitivity analysis was conducted using the Mantel-Haenszel method. Between-study heterogeneity was calculated using the Paule-Mandel estimator. Traditional meta-analysis statistics were performed using the ‘meta’ package in R (version 3.1.3; the R Project for Statistical Computing) with a P value <0.05 taken to indicate statistical significance. Adjusted ITCs of pooled estimates using inverse variance weighting were then performed according to the methods of Bucher and colleagues using the ITC computer program, Version 1.0 [E4-5 in S1 Appendix]. The likelihood of statistical heterogeneity was assessed using the I^2^ statistic, with a value >50% representing important statistical heterogeneity [E6 in S1 Appendix]. We planned to evaluate the presence of publication bias and related biases through visual inspection of funnel plots and Egger test for plot asymmetry, but the small number of studies limited the ability of these methods to detect meaningful effects.

## Results

**Fig 1:**
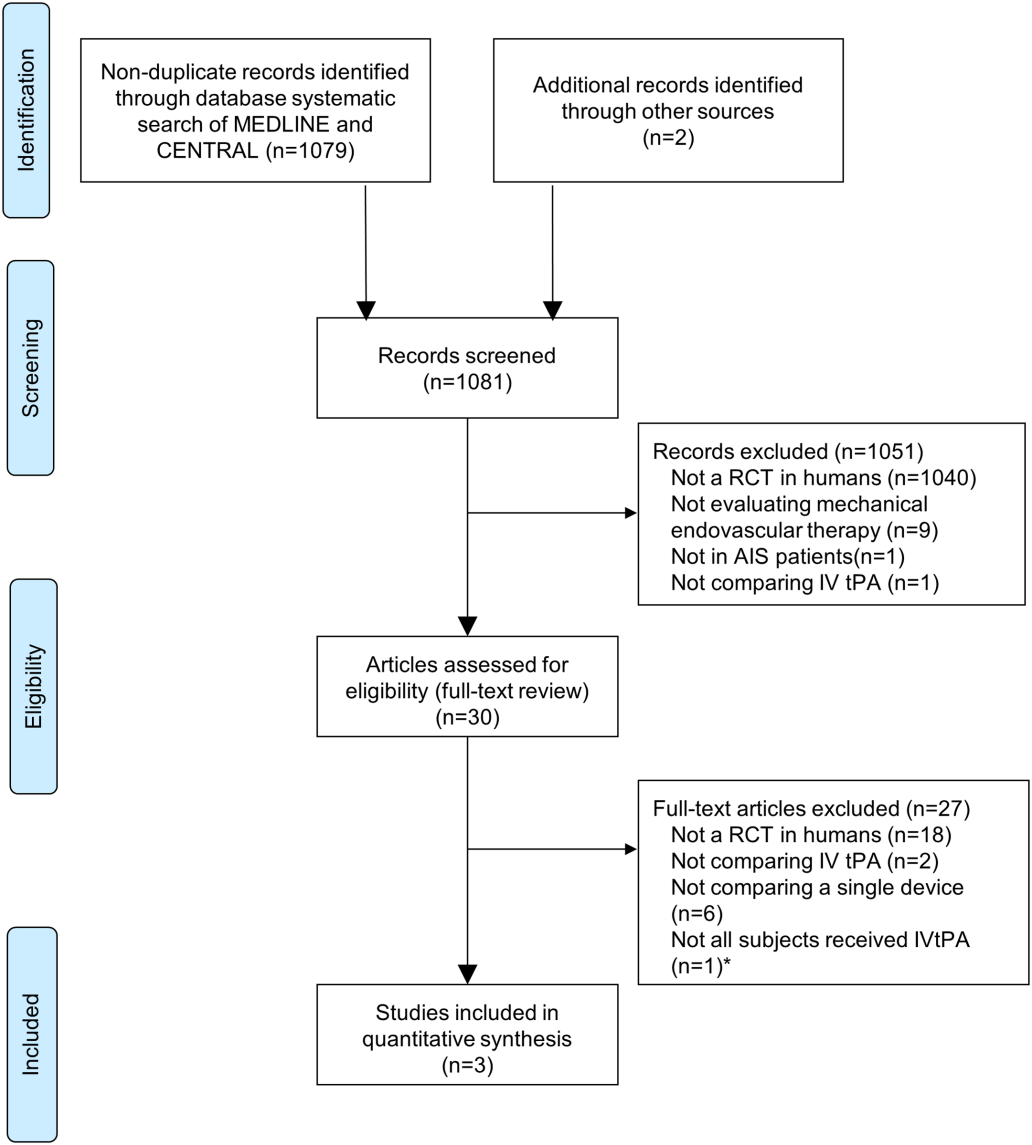
Flow diagram of study selection process for the systematic review and indirect treatment comparison. *REVASCAT met all other inclusion criteria, therefore it was included in the sensitivity analysis.

Three RCTs (n = 374 participants) were included in the primary analysis [1–3] (Fig 1). One additional study met all inclusion criteria, but did not administer IVtPA to the entire study population and was thus only included in the sensitivity analysis [4].

Characteristics of the included trials are detailed in Table 1. In each trial, neuroimaging-confirmed AIS patients received IVtPA ≤4.5 hours of symptom onset; those randomized to a thrombectomy device received the appropriate intervention ≤5–6 hours of symptom onset. The mean age range was 65–70 years; percentage of male patients 43–62%; and median NIHSS of 13–18.

**Table 1:** Baseline characteristics of patients in included randomized controlled trials.

Study, Year (N =) *	Trial Name	Intervention, (n)	Age, Mean ± SD	Male, n (%)	NIHSS, Median (IQR)	Stroke Onset to Groin Puncture In MET Intervention Group, Minutes, Median (IQR)
**Campbell 2015 (N = 70)**	EXTEND-IA	Solitaire+IVtPA (n = 35)	68.6 ±12.3	17 (49)	17 (13–20)	210 (166–251)
		IVtPA (n = 35)	70±11.8	17 (49)	13 (9–19)	
**Saver 2015 (N = 196)**	SWIFT PRIME^‡^	Solitaire+IVtPA (n = 98)	65.0 ± 12.5†	54 (55.1)^†^	17 (13–20)	224 (165–275)
		IVtPA (n = 98)	66 ±11.3†	45 (46.8)^†^	17 (13–19)	
**Mocco 2015 (N = 108)**	THERAPY^¶^	Penumbra+IVtPA (n = 55)	67.4±11.4	34 (61.8)	17 (13–21)	227 (184–263)
		IVtPA (n = 53)	70.1±10.3	23 (43.4)	18 (14–22)	
**Jovin 2015 (N = 206)**	REVASCAT^±^	Solitaire+IVtPA (n = 103)	65.7 ±11.3	55 (53.4)	17 (14–20)	269 (201–340)
		IVtPA (n = 103)	67 ±9.5	54 (52.4)	17 (12–19)	

IVtPA Intravenous tissue plasminogen activator; NIHSS = National Institute of Health stroke scale

*Intention to treat population

^†^Modified intention to treat population.

^‡^SWIFT PRIME: subjects were treated with either Solitaire Flow Restoration (FR) or Solitaire 2

^¶^THERAPY: subjects were treated with either Penumbra 3-dimensional (3D) Separator or Penumbra ACE aspiration catheter

^±^REVASCAT: not all subjects received IVtPA: n = 70 (60%) in the intervention group; n = 80 (77.7%) in the control (IVtPA) group

**Fig 2:**
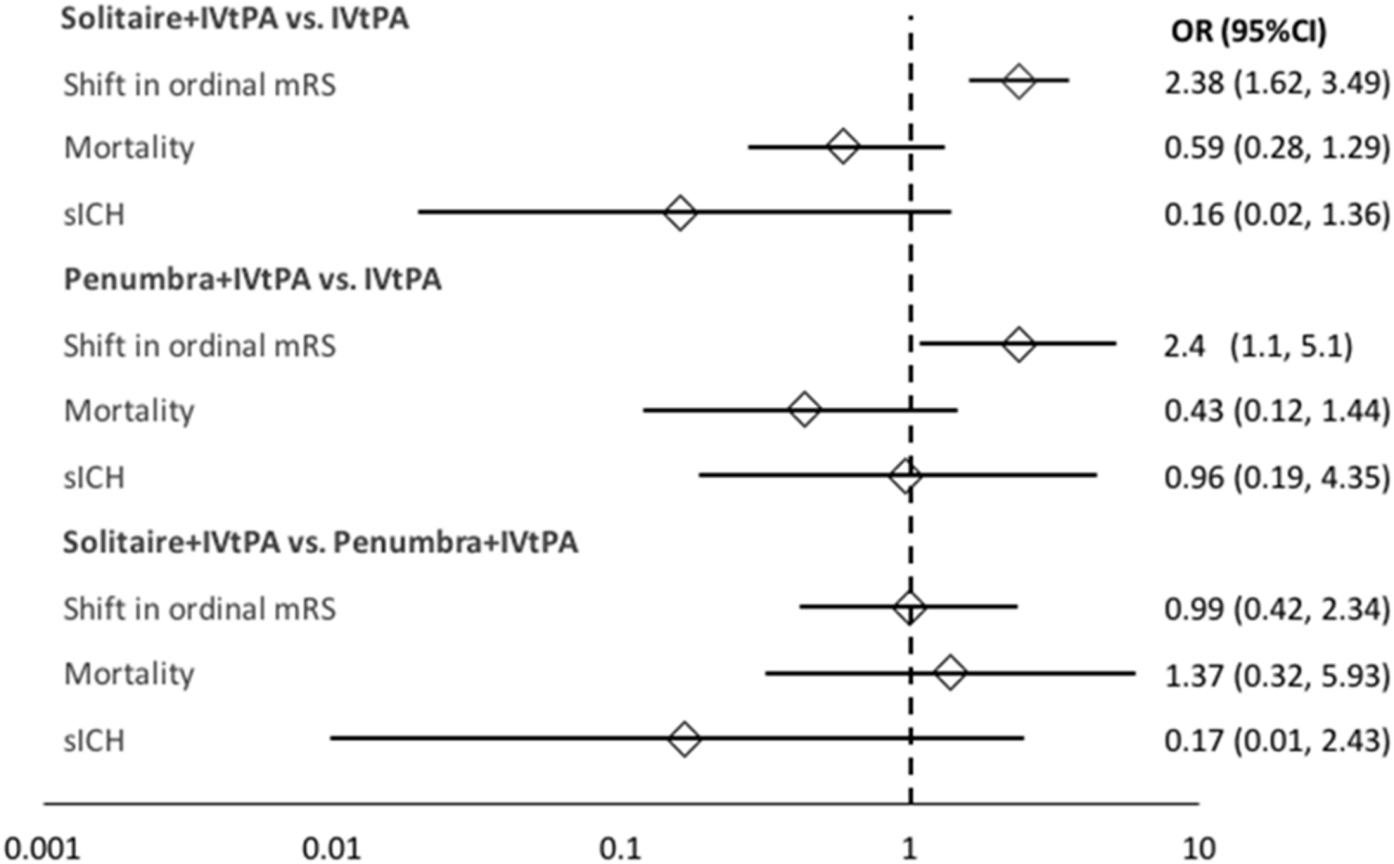
Results from the traditional and indirect treatment comparison meta-analyses. CI = 95% Confidence Interval; IVtPA = Intravenous Tissue Plasminogen Activator; mRS = modified Rankin Score; OR = Odds Ratio; sICH = Symptomatic Intracranial Hemorrhage.

Results are shown in Fig 2. Data from Stent-Retriever Thrombectomy after Intravenous t-PA vs. t-PA Alone in Stoke (SWIFT PRIME) and Endovascular Therapy for Ischemic Stroke with Perfusion-Imaging Selection (EXTEND-IA) were pooled using traditional meta-analytic methods to obtain the comparative effects of Solitaire stent retriever, finding that shift in ordinal mRS (OR, 2.38; 95% CI = 1.62–3.49) was significantly greater with thrombectomy [2–3]. The Aspiration Thrombectomy After Intravenous Alteplase Versus Intravenous Alteplase Alone (THERAPY) trial evaluated the Penumbra aspiration system, demonstrating a significantly greater shift in ordinal mRS (OR, 2.4; 95% CI = 1.1–5.1) compared to IVtPA alone [1]. No significant differences were observed in secondary outcomes. No significant statistical heterogeneity was seen for any endpoint (I^2^ = 0%). When the analyses were re-run using the Mantel Haenzel method, no appreciable differences in any of the outcomes were noted. Risk of Bias is reported in S1 Fig. It should be noted that THERAPY utilized two separate Penumbra devices, the 3-dimensional (3D) Separator as of December 2012 and the larger bore ACE aspiration catheter as of August 2013. All of the outcomes were pooled in the original study and are thus represented as such in our analysis.

**Fig 3:**
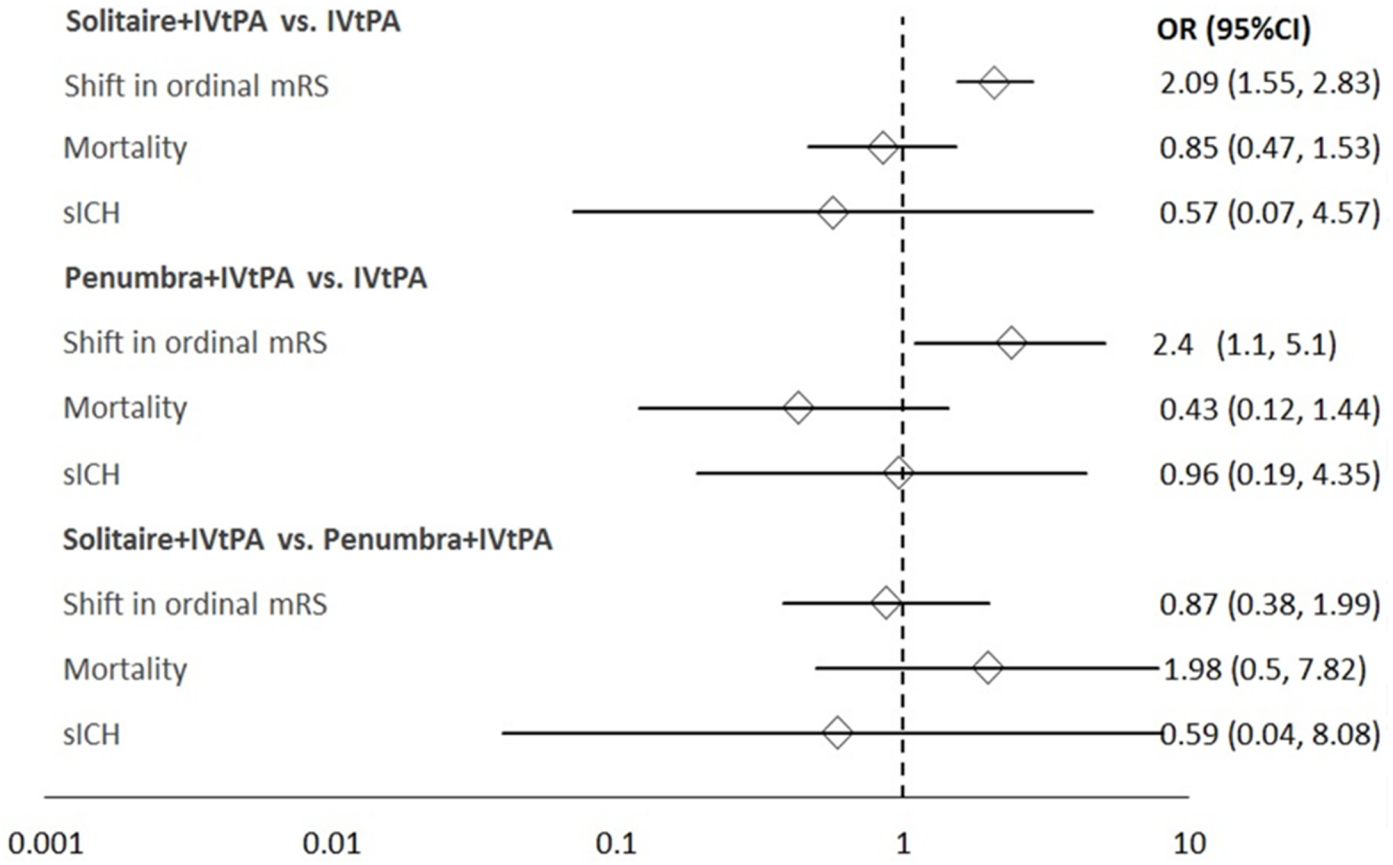
Sensitivity analysis: Results from the traditional and indirect treatment comparison meta-analyses incorporating the REVASCAT Trial. CI = 95% Confidence Interval; IVtPA = Intravenous Tissue Plasminogen Activator; mRS = modified Rankin Score; OR = Odds Ratio; REVASCAT = Thrombectomy within 8 Hours after Symptom Onset in Ischemic Stroke; sICH = Symptomatic Intracranial Hemorrhage.

Results from the ITC between Solitaire+IVtPA and Penumbra+IVtPA reveal no significant difference in outcomes, including shift in ordinal mRS (OR, 0.99; 95% CI = 0.42, 2.34). A sensitivity analysis incorporating the Thrombectomy within 8 Hours after Symptom Onset in Ischemic Stroke (REVASCAT) trial did not result in any significant changes to the primary efficacy or safety endpoints (Fig 3).

## Discussion

Traditional meta-analysis confirm that the devices Solitaire and Penumbra each improve efficacy outcomes while not affecting patient safety, with comparison between the two devices demonstrating no statistically-significant differences. While not significant, mortality at 90 days slightly favored Penumbra+IVtPA (OR 1.37; 95% CI = 0.32, 5.93), whereas sICH appeared to trend toward the Solitaire+IVtPA group (OR, 0.17; 95%CI = 0.01, 2.43).

Prior to MET, IVtPA was the mainstay of AIS treatment; however, its usage has many limitations including unresponsiveness of large thrombi to rapid enzymatic degradation, narrow time-window for administration and risk of systemic or ICH [3, 9]. Furthermore, >50% of patients receiving IVtPA have little improvement in disability or die [10].

A Randomized Trial of Intraarterial Treatment for Acute Ischemic Stroke (MR CLEAN) and Randomized Assessment of Rapid Endovascular Treatment of Ischemic Stroke (ESCAPE) were the first trials to show a benefit for MET with a stent retriever over IVtPA alone [5–6]. Four additional trials showed similar benefits, three using Solitaire and one with Penumbra [1–4]. However, it should be noted that the Therapy trial was stopped before primary endpoints were obtained due to lack of clinical equipoise. This implication is discussed further under the limitations of our study. While all studies demonstrated improved outcomes in AIS patients, the effects were not consistent across all trials. Notably, in RCTs with a shorter time from stroke onset to groin puncture, patients experienced higher rates of reperfusion and demonstrated greater odds of obtaining a favorable shift in mRS. Median time from stroke onset to groin puncture was ~265 minutes in MR CLEAN and REVASCAT, compared to 210–227 minutes in the EXTEND-IA, SWIFT PRIME and THERAPY trials. These latter three trials demonstrated higher rates of reperfusion (73–88%) and a more favorable shift in ordinal mRS (ORs = 2.1–2.67), compared to MR CLEAN and REVASCAT with reperfusion rates of 59% and 65.7%, respectively, and ORs = ~1.7 for ordinal shift in mRS. Future investigation may illustrate greater utility of MET in patients who undergo thrombectomy earlier in their clinical course.

While the results of implementing thrombectomy with IVtPA in AIS treatment has been overwhelmingly positive, concerns remain regarding device performance in heterogeneous populations. The studies in our analysis strictly excluded patients with larger, irreversibly ischemic cores and included those with protocol-defined measurements of salvageable ischemic tissue [2–4]. Additionally, two of the studies assessing the Solitaire stent retriever required neuro-interventionalists to complete ≥20 Solitaire procedures annually [3–4]; no such experience was required for Penumbra [1]. Finally, strict protocols were implemented to ensure patients received IVtPA ≤4.5 hours and MET ≤5–6 hours of symptom onset [1–4]. Future study is needed to determine if efficacy persists in patients with larger, irreversibly ischemic cores, who present 6 hours after symptom onset and undergo thrombectomy with less experienced neuro-interventionalists.

Finally, a review of the MET-device evidence is limited by the fact that the single Penumbra study (THERAPY) was halted early due to a lack of clinical equipoise secondary to external evidence. Despite being underpowered, THERAPY demonstrated a consistent suggestion of superiority for Penumbra, with the data indicating a benefit for the MET arm [1]. This, in turn, limits the current ITC to detect significant differences in outcomes when comparing Solitaire and Penumbra. One final limitation of the Therapy trial worth mentioning is that 13% (n = 7) of patients in the trial failed primary treatment with aspiration thrombectomy and were subsequently treated using either Solitaire or Trevo stent retriever. This may again limit the ability of our study to detect comparative efficacy between MET therapies.

Data from two ongoing prospective randomized trials may provide greater clarity in the comparative efficacy and safety of Solitaire and Penumbra MET devices; however, results are unavailable at this time [11–12]. In lieu of such randomized control trials demonstrating conclusive non-inferiority with aspiration thrombectomy, our meta-analysis serves to inform decision making by demonstrating equivalent efficacy and safety between Solitaire and Penumbra MET.

## Conclusion

Patients with AIS due to neuroimaging-confirmed large-vessel occlusion of the proximal anterior circulation, with small or moderate ischemic cores, exhibit significant improvements in efficacy parameters when treated with stent retrievers compared with IVtPA alone, without a significant change in safety outcomes. Indirect comparison of the Solitaire and Penumbra devices did not detect any significant differences in either efficacy or safety between the two devices. While awaiting the results of two prospective, randomized control trials, our analysis serves to inform clinical decision making regarding the treatment of AIS patients undergoing MET procedures.

## Supporting information

S1 Appendix References. (DOCX)

S2 Appendix Medline search strategy. (DOCX)

S1 Fig Risk of bias assessment of randomized controlled trials. +, low risk of bias;?, unclear risk of bias; -, high risk of bias.
(TIF)

S2 Fig PRISMA checklist. (PDF)

## References

[1] Mocco J, Zaidat OO, von Kummer R, Yoo AJ, Gupta R, Lopes D, et al;THERAPY Trial Investigators*. Aspiration Thrombectomy After Intravenous Alteplase Versus Intravenous Alteplase Alone. Stroke. 2016;47(9):2331–8. doi: 10.1161/STROKEAHA.116.013372 27486173 10.1161/STROKEAHA.116.013372

[2] Campbell BC, Mitchell PJ, Kleinig TJ, Dewey HM, Churilov L, Yassi N, et al; EXTEND-IA Investigators. Endovascular therapy for ischemic stroke with perfusion-imaging selection. N Engl J Med. 2015 3 12;372(11):1009–18. doi: 10.1056/NEJMoa1414792 25671797 10.1056/NEJMoa1414792

[3] Saver JL, Goyal M, Bonafe A, Diener HC, Levy EI, Pereira VM, et al; SWIFT PRIME Investigators. Stent-retriever thrombectomy after intravenous t-PA vs. t-PA alone in stroke. N Engl J Med. 2015 6 11;372(24):2285–95. doi: 10.1056/NEJMoa1415061 25882376 10.1056/NEJMoa1415061

[4] Jovin TG, Chamorro A, Cobo E, de Miquel MA, Molina CA, Rovira A, et al;REVASCAT Trial Investigators. Thrombectomy within 8 hours after symptom onset in ischemic stroke. N Engl J Med. 2015;372(24):2296–306. doi: 10.1056/NEJMoa1503780 25882510 10.1056/NEJMoa1503780

[5] Berkhemer OA, Fransen PS, Beumer D, van den Berg LA, Lingsma HF, Yoo AJ, et al; MR CLEAN Investigators. A randomized trial of intraarterial treatment for acute ischemic stroke. N Engl J Med. 2015;372(1):11–20. doi: 10.1056/NEJMoa1411587 25517348 10.1056/NEJMoa1411587

[6] Goyal M, Demchuk AM, Menon BK, Eesa M, Rempel JL, Thornton J, et al; ESCAPE Trial Investigators. Randomized assessment of rapid endovascular treatment of ischemic stroke. N Engl J Med. 2015 3 12;372(11):1019–30. doi: 10.1056/NEJMoa1414905 25671798 10.1056/NEJMoa1414905

[7] Powers WJ, Derdeyn CP, Biller J, Coffey CS, Hoh BL, Jauch EC, et al 2015 American Heart Association/American Stroke Association Focused Update of the 2013 Guidelines for the Early Management of Patients With Acute Ischemic Stroke Regarding Endovascular Treatment: A Guideline for Healthcare Professionals From the American Heart Association/American Stroke Association. Stroke. 2015 10;46(10):3020–35. doi: 10.1161/STR.0000000000000074 26123479 10.1161/STR.0000000000000074

[8] Health Quality Ontario. Mechanical Thrombectomy in Patients With Acute Ischemic Stroke: A Health Technology Assessment. Ont Health Technol Assess Ser. 2016 2 8;16(4):1–79. 27026799 PMC4761918

[9] Del Zoppo GJ, Saver JL, Jauch EC, Adams HP. Expansion of the time window for treatment of acute ischemic stroke with intravenous tissue plasminogen activator: a science advisory from the American Heart Association/American Stroke Association. Stroke. 2009;40(8):2945–8. doi: 10.1161/STROKEAHA.109.192535 19478221 10.1161/STROKEAHA.109.192535 PMC2782817

[10] Albers GW, Bates VE, Clark WM, Bell R, Verro P, Hamilton SA. Intravenous tissue-type plasminogen activator for treatment of acute stroke: the Standard Treatment with Alteplase to Reverse Stroke (STARS) study. JAMA. 2000 3 1;283(9):1145–50. 10703776 10.1001/jama.283.9.1145

[11] Lapergue B, Labreuche J, Blanc R, Barreau X, Berge J, Consoli A, et al ASTER Trial Investigators. First-line use of contact aspiration for thrombectomy versus a stent retriever for recanalization in acute cerebral infarction: The randomized ASTER study protocol. Int J Stroke. 2017 1. 10.1177/1747493017711948 28592218

[12] COMPASS Trial: a Direct Aspiration First Pass Technique (COMPASS). ClinicalTrials.gov. 2017 July 3. Cited 2017 July 5. https://clinicaltrials.gov/ct2/show/study/NCT02523261.

