# Supplementary material for: Mechanical endovascular therapy for acute ischemic stroke: An indirect treatment comparison between Solitaire and Penumbra thrombectomy devices
Source: PLoS One. 2018 Mar 7;13(3):e0191657. doi: 10.1371/journal.pone.0191657 (PMC5841644; doi:10.1371/journal.pone.0191657)
Supplement: S1 Appendix — (DOCX) [file pone.0191657.s001.docx]

**S1 Appendix: References**

1. Higgins JPT, Green S (eds). Cochrane Handbook for Systematic Reviews of Interventions Version 5.1.0 [updated March 2011]. The Cochrane Collaboration, 2011. Available at www.cochrane-handbook.org. Last accessed 27 June 2014.
2. Higgins JP, Altman DG, Gøtzsche PC, J€uni P, Moher D, Oxman AD et al. The Cochrane Collaboration’s tool for assessing risk of bias in randomised trials. BMJ 2011; 343: d5928.
3. DerSimonian R, Laird N. Meta-analysis in clinical trials. Control Clin Trials. 1986;7:177–188.
4. Bucher HC, Guyatt GH, Griffith LE, Walter SD. The results of direct and indirect treatment comparisons in meta-analysis of randomized controlled trials. J Clin Epidemiol. 1997;50:683–691.
5. Wells GA, Sultran SA, Chen L, Khan M, Coyle D. Indirect treatment comparison [computer program]. Version 1.0. Ottawa: Canadian Agency for Drugs and Technologies in Health; 2009.
6. Higgins JP, Thompson SG, Deeks JJ, Altman DG. Measuring inconsistency in meta-analyses. BMJ. 2003;327:557–560.
