# Supplementary material for: Mechanical endovascular therapy for acute ischemic stroke: An indirect treatment comparison between Solitaire and Penumbra thrombectomy devices
Source: PLoS One. 2018 Mar 7;13(3):e0191657. doi: 10.1371/journal.pone.0191657 (PMC5841644; doi:10.1371/journal.pone.0191657)
Supplement: S2 Appendix — (DOCX) [file pone.0191657.s002.docx]

| **S2 Appendix: Medline Search Strategy** | |
| --- | --- |
|  | Brain Ischemia/ |
|  | ((isch?emi* adj3 (stroke* or apoplex* or cerebr* or brain or encephalopath* or neur*)) or AIS) |
|  | Cerebrovascular Accident/ |
|  | Stroke/ |
|  | (stroke* adj3 (acute or cerebr* or attack* or accident* or lacunar or cardioembolic) |
|  | Occlusive Cerebrovascular Disease/ |
|  | Carotid Artery Obstruction/ |
|  | Carotid Artery Thrombosis/ |
|  | Intracranial Arteriosclerosis/ |
|  | “Intracranial Embolism and Thrombosis”/ |
|  | Brain Embolism/ |
|  | ((occlus* or block* or infarct* or clot* or termination) adj6 (carotid or cerebr* or MCA or ACA)) |
|  | or/1-13 |
|  | Mechanical Thrombectomy/ |
|  | Thrombectomy/ |
|  | Embolectomy/ |
|  | ((Mechanical adj3 (thromb* or embol* or clot disruption* or clot retrieval*)) or ((clot* or thromb* or embol*) adj3 (retriev* or disruption* or fragmentation)) or ((stent* or stent-assisted) adj3 retriev*) or stentriever*) |
|  | ((Merci or Trevo or Penumbra or Solitaire) adj3 (retriever* or system* or device*)) |
|  | or/14-18 |
|  | 13 and 19 |
|  | limit 20 to English language |
|  | limit 21 to humans |
|  | limit 23 to yr="2005 -Current" |
