# Supplementary figures and images for: Mechanical endovascular therapy for acute ischemic stroke: An indirect treatment comparison between Solitaire and Penumbra thrombectomy devices
Source: PLoS One. 2018 Mar 7;13(3):e0191657. doi: 10.1371/journal.pone.0191657 (PMC5841644; doi:10.1371/journal.pone.0191657)

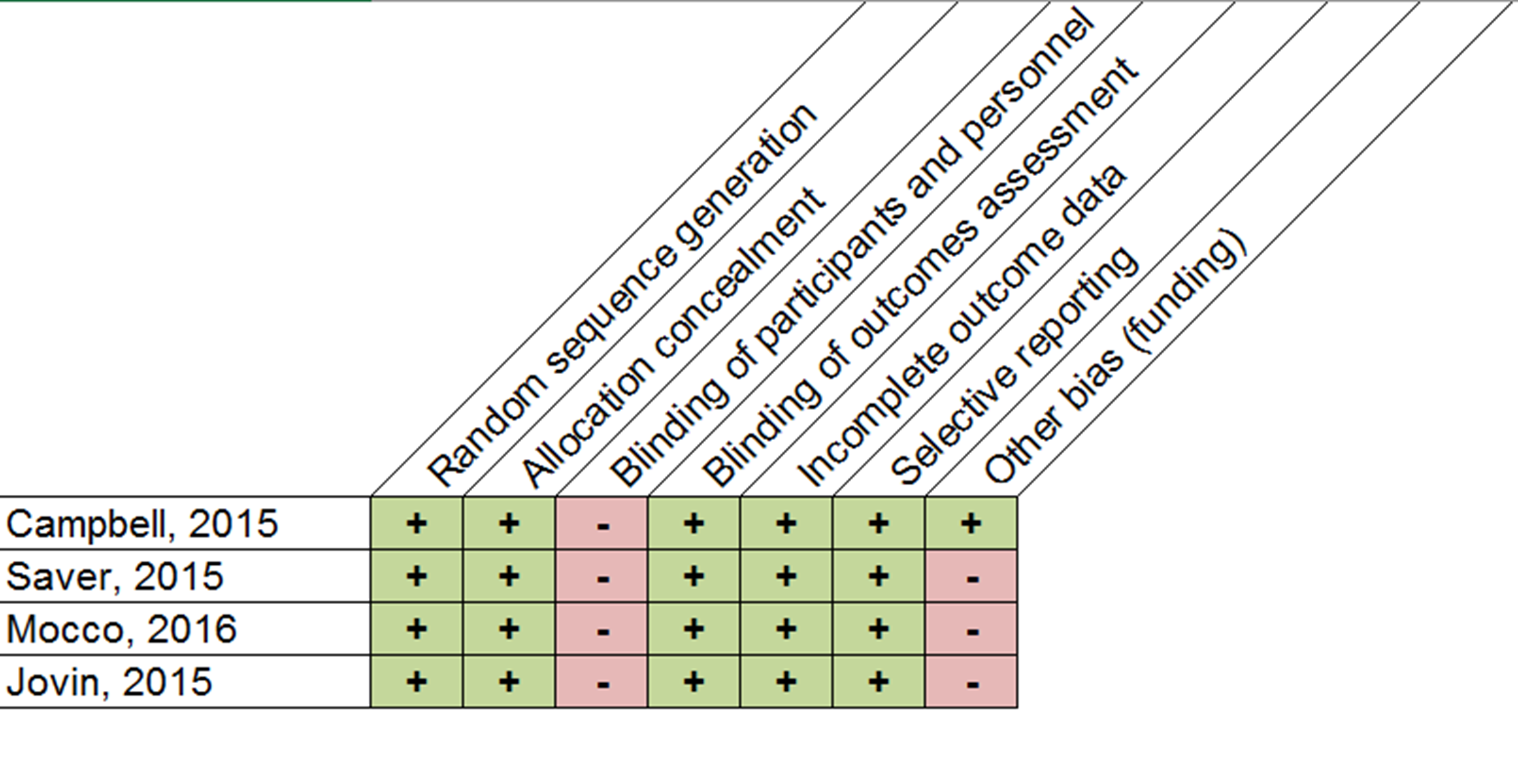

Supplement: S1 Fig — +, low risk of bias;?, unclear risk of bias; -, high risk of bias. (TIF) [file pone.0191657.s003.tif]
